# Droplet Microfluidics for High-Throughput Screening and Directed Evolution of Biomolecules

**DOI:** 10.3390/mi15080971

**Published:** 2024-07-29

**Authors:** Goran T. Vladisaljević

**Affiliations:** Department of Chemical Engineering, Loughborough University, Loughborough LE11 3TU, UK; g.vladisavljevic@lboro.ac.uk; Tel.: +44-(0)1509222518

**Keywords:** in vitro compartmentalization, droplet microfluidics, enzyme and RNA screening, microgels, giant unilamellar vesicles, emulsions

## Abstract

Directed evolution is a powerful technique for creating biomolecules such as proteins and nucleic acids with tailor-made properties for therapeutic and industrial applications by mimicking the natural evolution processes in the laboratory. Droplet microfluidics improved classical directed evolution by enabling time-consuming and laborious steps in this iterative process to be performed within monodispersed droplets in a highly controlled and automated manner. Droplet microfluidic chips can generate, manipulate, and sort individual droplets at kilohertz rates in a user-defined microchannel geometry, allowing new strategies for high-throughput screening and evolution of biomolecules. In this review, we discuss directed evolution studies in which droplet-based microfluidic systems were used to screen and improve the functional properties of biomolecules. We provide a systematic overview of basic on-chip fluidic operations, including reagent mixing by merging continuous fluid streams and droplet pairs, reagent addition by picoinjection, droplet generation, droplet incubation in delay lines, chambers and hydrodynamic traps, and droplet sorting techniques. Various microfluidic strategies for directed evolution using single and multiple emulsions and biomimetic materials (giant lipid vesicles, microgels, and microcapsules) are highlighted. Completely cell-free microfluidic-assisted in vitro compartmentalization methods that eliminate the need to clone DNA into cells after each round of mutagenesis are also presented.

## 1. Introduction

**Figure 1:**
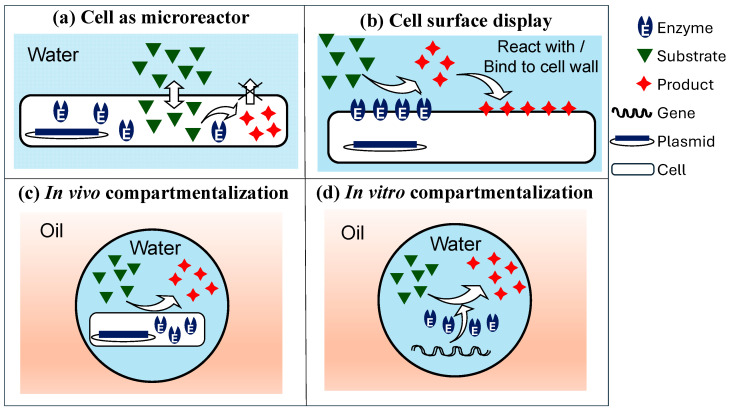
Typical HTS strategies based on fluorescence-based assays [6]. The droplet-based screening strategies shown in (**c**,**d**) will be discussed in this review.

The lack of rapid high-throughput screening (HTS) systems is a major bottleneck in creating biocatalysts via directed evolution [1] and functional genomics [2]. HTS is generally considered to include methods with screening rates between 10^4^ and 10^5^ per day, while ultra-HTS methods are characterized by screening rates above 10^6^ per day [3]. With advanced robotics, microtiter plates (MTPs) can screen libraries of up to 10^5^ clones per day [4], which is ~1/s, albeit at a high cost. The screening of single cells by fluorescence-activated cell sorting (FACS) allows for analysis of up to 50,000 cells per second [5]. However, FACS is only possible under two conditions: (a) the produced enzyme stays in the cytoplasm and the substrate is able to diffuse into the cell while the fluorescent reaction product is trapped inside the cell (Figure 1a), or (b) the enzyme is displayed at the cell surface and the fluorescent reaction product is also attached on the cell surface (Figure 1b). This is because in flow cytometers, the environment is controllable at a population level but uncontrollable and unmeasurable in close proximity to an individual cell.

Emulsion droplets can serve as miniaturized reaction vessels that can link genotype (the enzyme-encoding gene) and phenotype (catalytic activity) even if the reaction product is released from the cells (Figure 1c) or separated from the enzyme-encoding gene (Figure 1d). In this approach, the droplet boundary protects the genotype/phenotype linkage. This review will discuss the developments in using microfluidic chips for the directed evolution of biomolecules (enzymes and nucleic acids), with the main emphasis being on the various strategies for performing the on-chip fluidic operations involved in the process. We describe the key case studies for directed evolution and HTS within single and multiple emulsion droplets and biomimetic materials (GUVs and microgels).

### 1.1. Comparison of Compartmentalization in Droplets and Multi-Well Plates

Due to sub-100-micron droplet diameters, reaction volumes can be reduced from the microliter to the nano- and pico-litre ranges, which allows for screening at the level of single genes. For example, a droplet with a diameter of 10 μm has a volume of 0.5 pL, which is more than 10^8^ times smaller than the volume of each well in a typical 96-well plate (100–200 μL). Having a large number of small compartments is critically relevant for enzyme screening because there are 20^N^ possible combinations of amino acids in a protein molecule, which is N amino acids long, and enzymes typically contain 180–600 amino acids [3]. However, although droplets enable the screening of biomolecules in small volumes without capillarity or evaporation problems, the droplet interface is not completely impermeable, as the plastic or glass surfaces of test tubes and multi-well plates and, therefore, small molecules can diffuse between the droplets. The crosstalk can be minimized using suitable carrier oils and surfactants, providing a solid shell around droplets or solidifying the entire droplet volume. On the other hand, evaporation is more suppressed in droplets than in test tubes or microwells. A detailed comparison between microtiter plates, FACS, and droplet-based screening methods was reviewed recently [7]. 

### 1.2. Advantages of Microfluidic Platforms in HTS Applications

Microfluidic devices can achieve screening capacities above 10^7^ variants a day with kHz frequencies [8]. For a given flow rate of the dispersed phase, the screening frequency is inversely proportional to the droplet volume [9]. Other advantages of microfluidic workflows are (a) the high encapsulation efficiency of compartmentalized species because droplets are generated by applying very small shear forces; (b) the fact that several fluidic operations can be performed within the same chip at different locations; (c) the fact that multiple droplet generation junctions can be operated with a single set of pumps or pressure reservoirs to increase the throughput; (d) the fact that microchannel geometry can be re-designed quickly using computer-aided design (CAD) software and replicated with high precision during microfabrication; (e) the fact that the droplet size, morphology, and composition can be accurately controlled and re-adjusted in real time; and (f) the fact that the consumption of reagents and plasticware (tubes, plates, and pipette tips) is small due to automated system operation. Furthermore, droplets can be individually manipulated (fused with other droplets or continuous liquid streams, split into daughter droplets, incubated, probed, and sorted) with high precision, and the timing of each operation (mixing, reagent addition, assay reaction, sorting) can be controlled by the channel geometry, fluid flow rates, and applying local external forces, e.g., electrical or acoustic forces. Also, microfluidic channels are optically accessible, which allows for visual control over droplet production and manipulation and optical readout.

### 1.3. Bulk Emulsification versus Droplet Microfluidics

Droplets can be rapidly generated using traditional bulk emulsification methods. For example, a lab-scale ULTRA-TURRAX dispersing machine can generate more than 10^10^ droplets per minute (>6 × 10^11^/h). In microfluidic devices, droplets are produced continuously, but at much smaller rates, typically between 10^5^/h and 10^8^/h [10].

Furthermore, droplets generated using bulk emulsification methods show wide variations in size, with a typical coefficient of variation of 20–50%. In this case, a single gene or cell encapsulation is difficult to achieve since each twofold change in droplet diameter corresponds to an eight-fold change in volume, so large droplets dominate the total volume and are much more likely to be occupied by multiple cells. For example, the probability that each droplet will contain multiple cells will jump from 5% to nearly 78% if the droplet diameter is doubled at the same dilution. Droplet microfluidic chips can consistently generate droplets with a size variation of <3%, which satisfies the NIST criteria for monodispersity (“a particle distribution may be considered monodisperse if at least 90% of the distribution lies within 5% of the median size”). Indeed, the diameters of droplets produced in microfluidic devices are distributed normally around the mean value, so that 90% of the droplets have diameters lying within 1.64 standard deviations (σ) from the median diameter (D_50_) and, thus, 1.64σ ≤ 0.05D_50_ or σ/D_50_ ≤ 0.03, which means that CV ≤ 3%. Monodisperse droplets enable the more accurate quantification of the reaction product based on the optical readout compared to polydisperse droplets [8]. 

Moreover, double emulsion droplets with core–shell morphologies can easily be produced in microfluidic chips, which makes it possible to screen droplets using FACS. A two-stage bulk emulsification method typically results in double emulsion droplets, in which plenty of inner droplets are entrapped in a single outer droplet, and such inner droplets cannot be screened one-by-one because their projected areas will overlap as they flow down the screening channel. The main disadvantages of lab-on-a-chip devices are wetting and clogging problems due to small dimensions of microfluidic channels. As a result, lab-on-a-chip devices are often disposable, and a new chip must be fabricated for each HTS experiment. Also, microfluidic devices require more skillful operators than traditional screening platforms due to complicated start-up, shut-down, and operating procedures. 

## 2. Fabrication Methods for Microfluidic Devices

### 2.1. Soft Lithography

**Figure 2:**
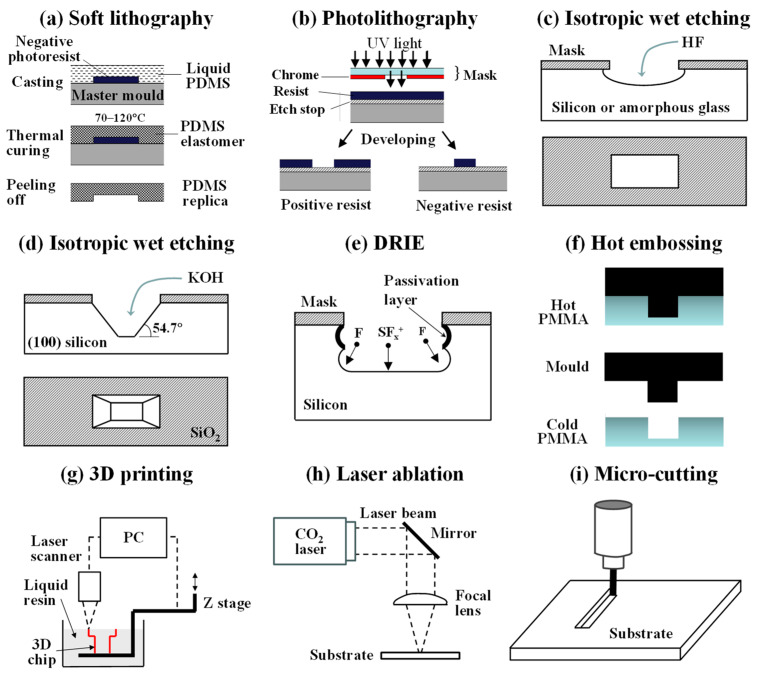
Fabrication methods for lab-on-a-chip devices.

Microfluidic chips can be made by soft lithography in polydimethylsiloxane (PDMS) using the protocol developed by Xia and Whitesides [11]. The entire fabrication process involves two main steps (Figure 2a): creating a silicon master mould by photolithography and forming microfluidic channels by replica moulding (Figure 2a). The photoresist can be patterned by passing ultraviolet light through a mask or by applying the projected light or focused beam of charged particles directly onto the resist surface without the need for a mask, which are known as optical maskless lithography and charged particle maskless lithography, respectively [12]. Although PDMS chips start to deform at pressures above 15 psi [13], can absorb small organic molecules from contacting fluids [14], and swell in contact with some organic solvents [15], they remain the most frequently used chips in biological applications due to their high compatibility with biological samples, low auto-fluorescence [16], high oxygen permeability [17], and inherent hydrophobicity, which can be reinforced using several techniques [18,19]. 

### 2.2. Photolithography and Etching

Traditionally, microfluidic chips have been fabricated using single-crystal silicon or glass substrates using manufacturing processes borrowed from the semiconductor industry. Glass chips can be manufactured by photolithography (Figure 2b) and isotropic wet etching with hydrofluoric acid, which results in rounded channels and the undercutting of the mask edges (Figure 2c) [20]. Reactive-ion etching and laser ablation can be used to create channels with vertical edges without undercutting, albeit at smaller etch rates [21]. Glass droplet microfluidic chips can be bought “off-the-shelf” from Dolomite Microfluidics. Unlike PDMS, glass is highly rigid, is resistant to organic solvents, and has superior optical properties and thermal stability, which allows for the regeneration of glass chips at high temperatures. Shallow channels with trapezoidal or triangular cross sections can be patterned in single-crystal silicon and quartz substrates by anisotropic wet etching using potassium hydroxide (Figure 2d) or tetramethylammonium hydroxide [22], while deep reactive-ion etching (DRIE) (Figure 2e) is used to etch deep narrow structures in silicon [23]. Silicon chips are expensive to fabricate, brittle, and non-transparent to visible light, so channels must be observed by metallurgical (reflective) microscopes. 

### 2.3. Hot Embossing and Injection Moulding

Chips from thermoplastics such as poly(methyl methacrylate) (PMMA), cyclic olefin copolymer (COC), polystyrene (PS), and polypropylene (PP) can be fabricated using hot embossing (Figure 2f) and injection moulding. Hot embossing involves pressing a mould into a softened polymer. Once the polymer has conformed to the shape of the mould, it is cooled below the glass transition temperature so that it is sufficiently hard to separate from the mould. Injection moulding is the process by which a softened polymer is introduced into a mould cavity under pressure. Both techniques are suitable to produce microfluidic devices for cell-based assays rapidly and cost-effectively [24,25].

### 2.4. Three-Dimensional Printing

Three-dimensional printing has become increasingly popular in recent years [26] due to the decreasing costs of 3D printers, an increase in printing resolution, and the ability to create true 3D channels. Fused deposition modelling (FDM) 3D printing involves the injection of a hot polymer melt through a nozzle onto an XYZ stage to build up a three-dimensional structure layer-by-layer. These layers then rapidly cool and solidify. The advantages of FDM 3D printing include the ease with which different materials can be switched during printing, biocompatibility, and the wide range of materials available for printing. The main drawbacks are related to relatively poor resolution (50 μm or above) and inferior surface smoothness compared to other 3D printing methods [27,28]. SLA (stereolithography) and DLP (digital light processing) 3D printers build up material through the polymerization of a liquid photopolymer using a guided laser beam (SLA) or a projector (DLP) (Figure 2g). DLP 3D printers use a digital projector screen to flash an image across the entire layer, curing all points at the same horizontal position simultaneously, while SLA printers cure one spot at a time [29]. Three-dimensional printing has the potential to become the method of choice for the microfabrication of lab-on-a-chip devices, especially for bioanalysis [30]. 

### 2.5. Laser Ablation and Microcutting

Laser ablation (Figure 2h) is a process in which a laser beam removes material through thermal vaporization or molecular disintegration. It can be used for the complete fabrication of microfluidic devices in several hours, including the maskless generation of microfluidic patterns on substrates such as epoxy resin [31], poly(methyl methacrylate) (PMMA) [32], poly(vinyl chloride) (PVC), polycarbonate (PC) [33], and glass [34,35]; drilling inlet and outlet ports; and bonding two plates together to enclose the generated patterns and make the device leak-proof [34,35]. 

Micro-cutting (Figure 2i) is based on removing bulk material using drilling and milling micro-cutting tools. Drill diameters less than 25 μm are available, and the tool speed and position can be controlled by CNC. Micro-milling can easily manufacture 3D structures with high aspect ratios [36] and multi-height features, which cannot easily be manufactured by photolithography. A wide range of materials can be milled, such as metals, polymers, composites, and ceramics. Stainless steel is highly advantageous for the fabrication of master moulds due to high wear resistance.

### 2.6. Glass Capillary Pulling

Glass capillary microfluidics is based on using coaxial assemblies of borosilicate glass capillaries to manipulate fluids and generate droplets. Typically, tapered-end inner capillaries are prepared by pulling commercially available glass capillaries with an outer diameter of about 1 mm, followed by tip cutting or sanding [37].

## 3. Microfluidic Unit Operations for Directed Evolution of Biomolecules

Basic on-chip operations performed during the directed evolution of biomolecules are reagent mixing, droplet generation, droplet incubation, and sorting.

### 3.1. On-Chip Reagent Mixing

#### 3.1.1. Merging Two Continuous Fluid Streams

**Figure 3:**
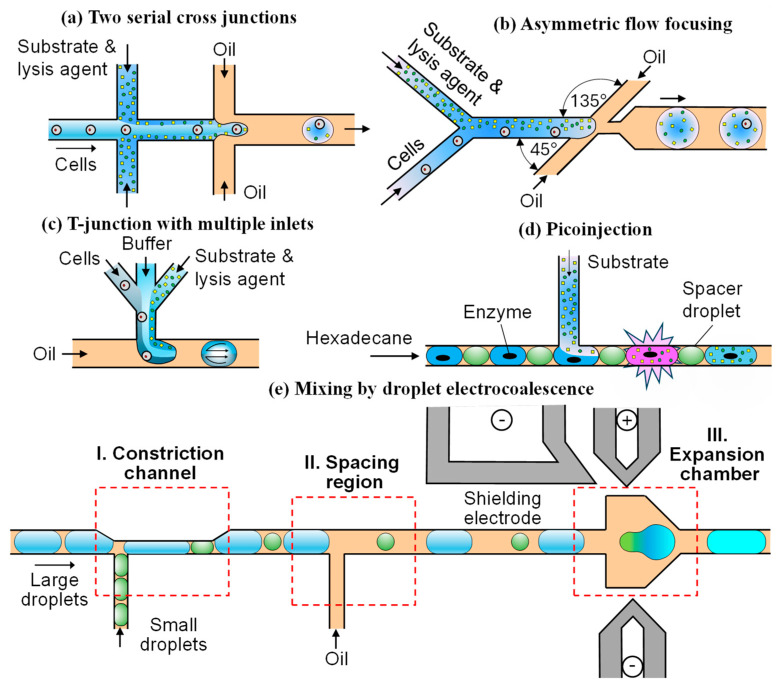
Main strategies for on-chip mixing of cells and reagent solutions.

The on-chip mixing of cells and reagents in directed evolution is carried out shortly before or after droplet generation, which prevents premature assay reaction. In the case of two serial cross junctions, as shown in Figure 3a [38,39], cells suspended in a growth medium are injected through the main channel, while the substrate and lysis agents are delivered through two upstream side channels. The mixing ratio of reagents can be varied in real time by changing the flow rate ratio of inlet streams. The combined stream is flow-focused by a carrier oil in the downstream junction and broken up into droplets. Mixing in a straight channel between the two junctions is very slow and carried out by diffusion due to laminar flows. The mixing efficiency in this region can be improved by incorporating chevron-shaped (herringbone) grooves on the top and/or bottom of the channel [40,41,42,43]. In the Stokes flow regime, these grooves cause lateral perturbations in the channel, resulting in two counter-rotating vortices across the channel and more vigorous mixing.

The process of droplet generation can be divided into three main stages: filling, necking, and pinch off. During the filling stage, the velocity at the interface between two liquid phases is much smaller than the velocity of the continuous phase. Thus, the flow of the continuous phase induces vortices in the dispersed phase during droplet formation. In a flow-focusing droplet maker, as shown in Figure 3b [43], the orifice inserted between the side channels and the outlet channel increases the difference in velocity between the two phases to enhance the impact of these vortices on the mixing process. The symmetry of the side channels determines the symmetry of the vortices and the mixing efficiency. The asymmetric design shown in Figure 3b with side channels arranged at 45° and 135° to the central channel enables the formation of a single asymmetric recirculation vortex in the dispersed phase during the droplet formation stage, which leads to faster mixing, compared to the symmetric design characterized by two symmetric vortices [43]. The rapid mixing of reagents is important for precise enzyme kinetic measurements. 

Substrate and cells can be injected into the microfluidic device through a T junction with two converging inlet arms [44]. The cell suspension is delivered through one arm and the fluorogenic substrate through the second symmetrical arm. The two streams meet at the junction and form droplets in a cross-flowing oil phase. The two streams can be separated by a buffer stream in the middle to prevent premature mixing and achieve the controlled dilution of the mixture inside droplets, as shown in Figure 3c [45]. In a straight downstream channel (Figure 3c), two symmetric vortices form in the upper and lower halves of a newly formed droplet. The mixing occurs by convection within each vortex and by diffusion between the two vortices [45]. The mixing within moving droplets can be enhanced by replacing straight channels with winding (serpentine) channels in which the interface between the two recirculating halves of the droplet is subjected to repeated stretching, folding, and reorienting events, facilitating the mixing process [46]. The most efficient mixing in winding channels occurs when the droplet diameter is comparable with the channel width [47]. The mixing inside droplets can be additionally enhanced by introducing bumps into winding channels that induce an abrupt variation in channel width [48].

#### 3.1.2. Merging a Continuous Fluid Stream and a Droplet

Picoinjection (Figure 3d) involves on-chip injection of a reagent solution flowing through a side channel directly into pre-formed droplets. It was first applied to prepare an enzyme-inhibitor assay by injecting a substrate solution into the droplets of a buffer solution containing enzyme and inhibitor reagents [49]. Perfluorodecalin (PFD) droplets acted as spacers between the enzyme droplets to prevent their coalescence. Both the enzyme and PFD droplets were alternately formed in a continuous stream of hexadecane and passed by the T junction, where the substrate solution was introduced into each droplet. If the droplets are stabilized by surfactants, picoinjection can be triggered by an electric field [50]. In this case, the volume injected into each droplet can be controlled with sub-picoliter precision at kilohertz rates. When the electric field is not activated, a surfactant layer adsorbed at the drop surface prevents the substrate solution from entering the drops. When the electric field is activated, the interface is ruptured and the droplet and substrate solution merge. This strategy is frequently used in directed evolution of biomolecules to inject a fluorogenic substrate into droplets containing either cells or in vitro transcription mixture [51]. Serial and combinatorial injections within a single droplet can be achieved by using multiple picoinjectors in series, each separately controlled by an electric field [52,53]. However, the utilization of an electric field can be harmful to encapsulated cells or biomolecules. Electricity-free picoinjection techniques have been developed to inject reactants into surfactant-stabilized droplets by precisely controlling the pressures inside a microfluidic device [54]. 

#### 3.1.3. Merging Two Droplets

An alternative way of performing on-chip mixing is based on pairing and merging two sets of reinjected droplets, each carrying a different aqueous phase. This approach requires the precise synchronization of the injection frequencies of two droplet sets, which is challenging to achieve, especially for differently sized droplets. The droplet merging method shown in Figure 3e enables the self-synchronization of reinjected droplets and high-efficiency droplet pairing and merging even when the droplet sizes differ significantly [55]. The chip consists of three parts: the constriction channel for droplet synchronization, the spacing region for droplet separation, and the expansion chamber for droplet coalescence. Two sets of droplets with different sizes are reinjected into separate intersecting channels. When a large droplet flows into the constriction channel, the side channel is temporarily blocked, preventing the next small droplet from entering the main channel. When the large droplet passes through, the side channel becomes open, causing the small droplet to quickly follow the large droplet and enter the main channel. Likewise, the small droplet temporarily obstructs the main channel and stops the flow of large droplets. After the small droplet passes through, the next large droplet enters the main channel, starting the next cycle of droplet arrangement. Upon synchronization, the pairs of droplets flow through another T-junction, where an oil phase is introduced from the side channel to increase the gap between adjacent droplets. As the pairs of droplets enter the expansion chamber, the velocities of both droplets significantly decrease, causing them to come into contact with one another. The top left electrode serves as the shielding electrode to prevent droplet coalescence during reinjection and arrangement, while the top right and bottom electrodes are used to trigger droplet coalescence. This technique can be applied to pair two single cells into droplets for cell fusion and cell–cell interaction studies, as well as to pair single cells and single hydrogel beads for single-cell barcoding and sequencing [55]. 

Several other methods have been tested for droplet pairing including the hydrodynamic coupling of two T-junction droplet generators [56], cleaving an aqueous stream by a water-in-oil emulsion droplet to generate a second water-in-oil emulsion droplet [57], and combining droplet railing and floating trap arrays [58]. 

### 3.2. Droplet-Based Sample Compartmentalization

**Figure 4:**
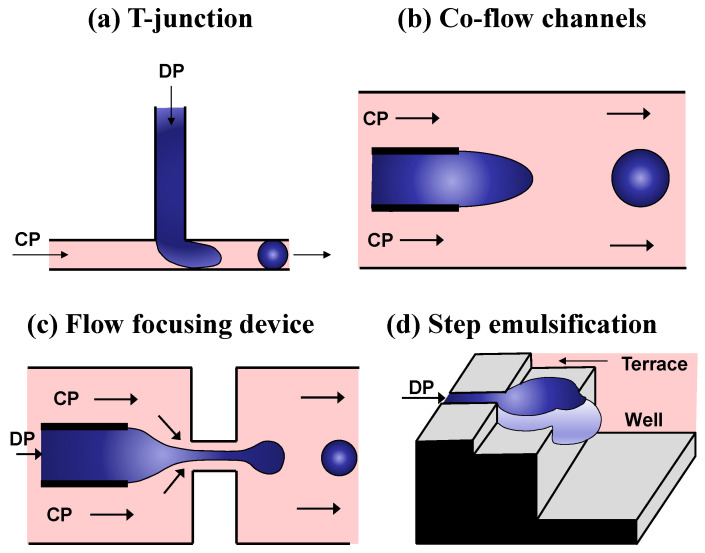
Main microfluidic strategies of droplet formation (DP = dispersed phase; CP = continuous phase). Adapted from [82].

Droplet generation units for generation of single emulsions are based on crossflow (T-junctions) [59], co-flow [60], elongated flow (flow-focusing) [61], and step emulsification [62] (Figure 4). In directed evolution studies, the crucial requirements for this operation are that droplets are formed in the dripping regime and a single-cell encapsulation is achieved, i.e., droplets must be monodispersed and “monoclonal”. 

**T-Junction:** In a T-junction, the continuous phase (CP) flows within the main channel, and the dispersed phase (DP) is injected from a side channel intersecting the main channel at 90° (Figure 4a). Droplets are generated due to shear forces induced by the continuous phase [59,63,64] or pressure force if the main channel is obstructed by the dispersed phase [65]. A T-junction is rarely used for droplet generation in HTS applications due to its low production frequency (typically less than 100 Hz) and large droplet sizes. 

**Co-flow droplet maker:** Coflowing microfluidic droplet generators consist of two coaxial channels, for example, a smaller capillary tube placed inside a larger tube (Figure 4b). The inner capillary supplies the DP, whilst the outer tube delivers the CP fluid [60,66]. Although this maker is rarely used in HTS due to large droplet sizes, when combined with counter-current flow-focusing, it allows the facile generation of multiple emulsions [67]. 

**Flow-focusing junction:** In this geometry, the DP flows in the middle channel and is enveloped by the CP coming from either side (Figure 4c). The two co-flowing liquid streams are usually forced through a small orifice downstream of the junction, and droplets are formed by viscous stress exerted on the inner phase by the surrounding outer phase flow. The geometry of these devices can vary from completely 2D (planar) [61] to quasi 3D [68,69] and axisymmetric 3D [70,71]. Axisymmetric devices deliver the DP through a cylindrical inlet channel, which is hydrodynamically focused by the CP as both phases pass through a circular baffle/constriction. Flow-focusing is the most frequently used method for droplet generation in directed evolution studies due to the high production frequency (>10 kHz in some cases), stable droplet generation process, and small droplet sizes that can be significantly smaller than the channel width [72,73]. 

**Microfluidic step emulsification:** Step droplet generators consist of a shallow and narrow microchannel delivering the DP and an abrupt (step-like) opening to a deep and wide reservoir (“well”) filled with the CP. The upstream narrow microchannel can terminate directly at the well [62], or a wide terrace can be added between the narrow channel and the well to reduce the flow velocity of the DP [74]. Inertial forces in the DP can also be minimized by delivering the DP through wedge-shaped channels [75,76]. 

In step emulsification, the droplet size in the dripping regime is independent of fluid flow rates, since the droplet pinch-off occurs due to differences in Laplace pressure between the droplet head and the neck and not due to shearing forces [62,77] (Figure 4d). As a result, step droplet generators can easily be parallelized by connecting many parallel shallow channels to the same terrace and the well [78]. The well can contain a stagnant [74] or crossflowing [78] CP liquid. In addition, narrow channels can be fabricated on the substrate surface as grooves [79] or across the cross section as straight-through holes [80]. Step emulsification was used in directed evolution studies to evolve RNA ligase that could resist inhibition by neomycin [81].

### 3.3. Droplet Incubation

Droplet incubation can last from several minutes to several weeks and can be performed on-chip or in a test tube [1,39]. The residence time t of droplets moving through a rectangular channel with a width of w and a height of h is

(1) $$t=\frac{L}{U}=\frac{Lwh}{Q}$$

where Q is the emulsion flow rate, L is the total channel length, and U is the average flow velocity. Therefore, the on-chip residence times of droplets can be increased either by increasing L or by decreasing U. 

#### 3.3.1. On-Chip Droplet Incubation in Serpentine Channels

**Figure 5:**
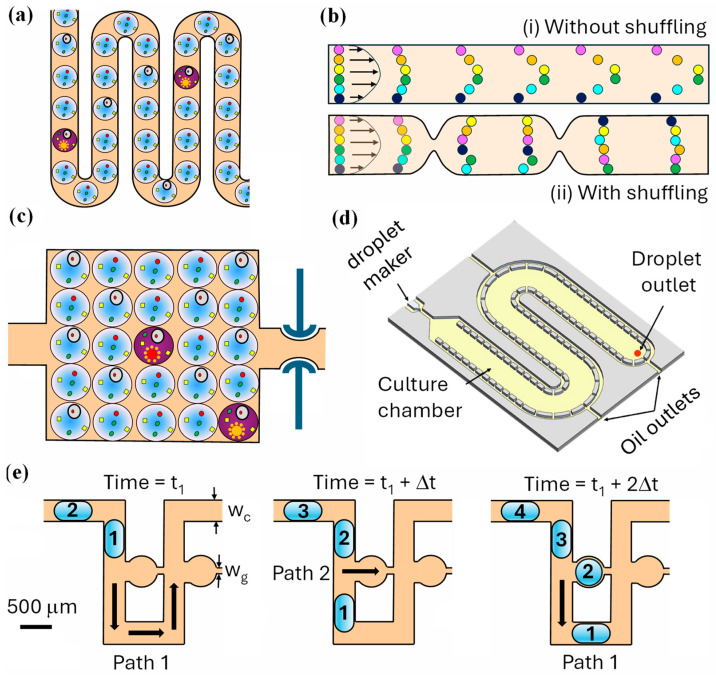
Strategies for on-chip droplet incubation: (**a**) serpentine channel [90]; (**b**) wide channel with and without constrictions [84]; (**c**) rectangular chamber with a pneumatic valve [85]; (**d**) wavy chamber with pillars along the side walls to remove the excess oil [86]; (**e**) flow resistance-based microwells for trapping droplets [87].

Serpentine channels (Figure 5a) increase the incubation time by extending L, with an added benefit being that droplet contents are mixed during the incubation period due to inertial forces at the turning points, especially when the undeformed droplet diameter is comparable to or larger than the smaller channel dimension [83]. However, the pressure drop along the channel is given by

(2) $$∆P=\frac{12\eta LQ}{wh^{3}}{\left[1-\frac{192}{\pi^{5}}\frac{h}{w}\tanh\left(\frac{\pi w}{2h}\right)\right]}^{-1}$$

where h is the smallest channel dimension (usually the channel height), and η is the emulsion viscosity. To obtain the residence time of just 1 min at Q = 0.5 mL/h, w = 50 μm, h = 40 μm, and η = 1 mPa·s, L must be 4.2 m and ∆P must be 42 bar. Both ∆P and L can be reduced by increasing w and h [9,84], since ∆P is inversely proportional to $wh^{3}$ and L is inversely proportional to wh for constant incubation time. For example, at w = 1000 μm and h = 75 μm, the required channel length for the same residence time is 0.11 m, and ∆P is 0.46 kPa. However, due to the parabolic velocity profile in the channel, the velocity in the centre of the channel is higher than the velocity near the walls. Therefore, the droplets in the centre move faster than the droplets close to the walls, leading to significant differences in the incubation times of individual droplets (Figure 5bi) [84]. The droplet residence time can be made more uniform by introducing constrictions along the incubation channel (Figure 5bii) that reduce the channel width to the droplet diameter and result in mixing droplets over the channel cross section as they flow down the incubation channel [84]. 

#### 3.3.2. On-Chip Droplet Incubation in Large Chambers

Wide incubation channels can evolve into chambers that may be 10,000 μm wide with storage capacities greater than 20,000 droplets. A pneumatic valve (Figure 5c) was used to decouple droplet generation from the incubation step [85]. During droplet generation, the pneumatic valve was opened, and the generated droplets were collected in the chamber. Once the chamber was filled with droplets, the valve was closed, and the droplets were incubated for the required period. The valve allowed for an oil phase to flow through while preventing droplets from escaping the chamber. It is a useful feature because longer incubations without continuous oil flow would lead to the complete removal of oil through PDMS walls and destabilization of droplets. 

Based on Equation (1), the incubation times of droplets in a chamber can be increased by reducing the emulsion flow rate Q, which is equal to $Q_{c}+Q_{d}$. One strategy to reduce Q in a culture chamber without compromising the droplet throughput is to extract oil from the emulsion during incubation. In this approach, $Q_{c}$ is high during droplet generation to maintain the dripping regime and has a low value during droplet incubation to prolong the incubation time. The device shown in Figure 5d combines a winding incubation chamber with an array of micro-pillars along the side walls to extract carrier oil. The droplets remain trapped inside the chamber because the gap between the pillars is smaller than the droplet diameter, resulting in a long residence time (1.5–4 days) [86]. 

#### 3.3.3. On-Chip Droplet Incubation in Trapping Microwells

Droplets can be trapped by microwells to be stationary and isolated from other droplets during incubation, as shown in Figure 5e. The two possible paths for incoming droplets are labelled as Path 1 and Path 2, and their flow resistances are R_1_ and R_2_, respectively. At the time t = t_1_, droplet 1 will bypass the well and follow Path 1 since R_1_ < R_2_, because the main channel is much wider than the trap outlet ($w_{c}$ » $w_{g}$). At t_1_ + Δt, the main channel is obstructed by droplet 1 and R_2_ < R_1_. Therefore, droplet 2 will follow Path 2 and be trapped. At t_1_ + 2Δt, since the well is occupied by droplet 2, droplet 3 will follow Path 1 and eventually be trapped by the next trap [87]. To avoid pushing the trapped droplet through the outlet, the pressure difference across the droplet interface should be below the critical pressure ($∆P_{c}$), as given by the Young–Laplace equation [88]:

(3) $$∆P_{c}=2\gamma \left(\frac{1}{h}+\frac{1}{w_{g}}\right)$$

where γ is the interfacial tension and h is the height of the trapping channel. The chip based on traps shown in Figure 5e was used to immobilize 180 droplets encapsulating *Caenorhabditis elegans* to enable the characterization of the animal behaviour in response to neurotoxins. Other microfluidic systems for the hydrodynamic trapping of droplets in microwells are also available [89]. 

### 3.4. Droplet Sorting

#### 3.4.1. Signal Detection

Droplet sorting includes signal detection followed by droplet actuation based on the intensity of the target signal. Various label-based and label-free detection methods have been used to sort droplets in microfluidic chips [91,92]. Label-based detection relies on the properties of labels (tags) for detecting a particular target. Fluorescence-activated droplet sorting (FADS) and absorbance-activated droplet sorting (AADS) are the two most common labelled detection methods based on fluorogenic and chromogenic assays, respectively. A microfluidic workflow with AADS was used to evolve phenylalanine dehydrogenase with improved activity toward its native substrate, solubility, and stability [93].

FADS is more sensitive than AADS (nM vs. high μM detection limits, respectively), but AADS is a simpler and cheaper method than FADS, as no excitation lasers and photomultiplier tubes are needed [94]. Also, FADS is typically 10–20 times faster than AADS (1–2 kHz vs. 0.1–0.3 kHz), since the absorbance is directly proportional to the length of the light path, meaning that larger droplets are needed for AADS. The throughput of AADS can be increased using refractive index matching oil and improved chip design [94]. 

Although optical detection is the gold standard for assaying enzymatic activities in droplet microfluidic chips, detection techniques for the structural characterization of compounds can be also used, including nano-electrospray ionization mass spectrometry (nano-ESI MS) [95], matrix-assisted laser desorption/ionization mass spectrometry (MALDI-MS) [96], surface-enhanced Raman scattering (SERS) [97], and nuclear magnetic resonance (NMR) [98]. In addition, the glycolytic activity of live cells encapsulated in droplets can be measured passively based on pH-dependent droplet deformability [99]. 

#### 3.4.2. Droplet Actuation

**Figure 6:**
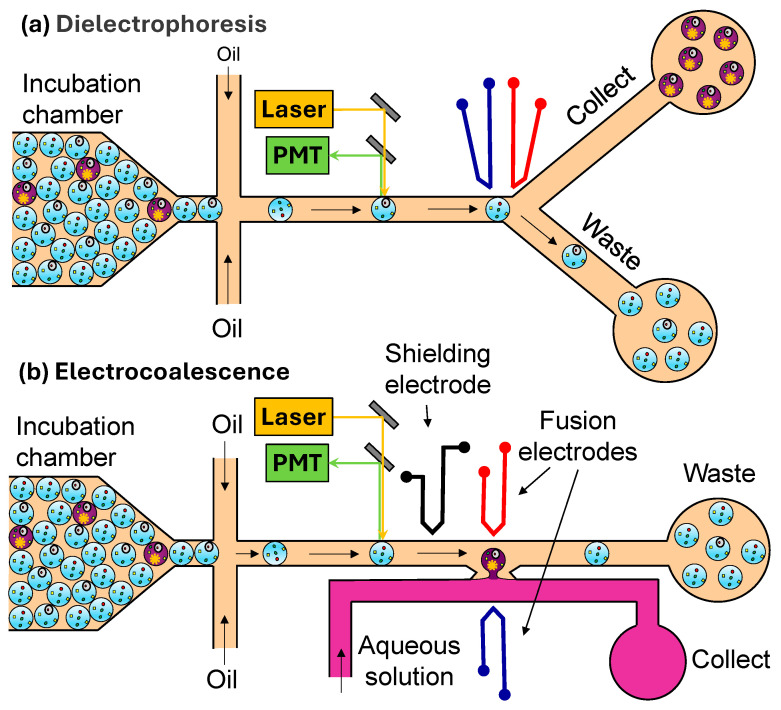
Fluorescence-activated droplet sorting (FADS) in microfluidic chips based on the following phenomena: (**a**) dielectrophoresis (DEP); (**b**) electrocoalescence [101].

Droplet actuators convert various forms of energy into mechanical motion of droplets. Droplet actuation can be accomplished using pneumatic, magnetic, thermal, acoustic, and electric (usually electrophoretic and dielectrophoretic) actuators. They work by pulling or pushing the desired droplets from a train of droplets into the hydrodynamically less favoured collection channel. The most widely used actuation method is dielectrophoresis (DEP), where target droplets are displaced from their original trajectories by a non-uniform electric field that creates a dipole moment on aqueous droplets dispersed in oil [100]. In a typical DEP droplet sorter (Figure 6a), the incubated droplets flow to a junction, where oil is added to separate the droplets before they are illuminated by a laser light of a specific wavelength (or defined wavelength band). The fluorescence of each droplet is measured with a photomultiplier tube (PMT). If the signal is higher than the threshold level, a pulse of an AC electric field is applied across the electrodes, forcing the droplet into the narrower/longer arm of the Y junction. Without an electric field, the droplets will not be displaced toward the electrodes, and they will all flow into the wider/shorter arm of the Y junction, which offers lower hydrodynamic resistance than the collection channel.

DEP is highly efficient for sorting water-in-oil droplets due to the high dielectric contrast that exists between water (~80) and oil (~2 to 6). It can be used for sorting droplets with volumes ranging from 20 fL to 10 nL at sorting rates up to 30 kHz, which is comparable with the rates achieved by commercial flow cytometers [102]. DEP has multiplexed sorting capabilities and can be used to sort up to five different droplet populations simultaneously [90]. DEP is not restricted to fluorescence-based detection but can also be performed through absorbance or morphological measurements [96]. 

Fluorescence-activated electrocoalescence (FAE) is an alternative method of droplet sorting [97], which allows the direct extraction of droplet contents into an aqueous stream without the need to break sorted droplets off-chip. As shown in Figure 6b, the aqueous stream and the oil stream containing droplets are co-flowed between two electrodes. In the absence of an electric field, the droplets are transferred into the waste line without coalescence. When a droplet with a fluorescence level over a pre-defined threshold is detected, a pulse of an AC electric field is applied across the electrodes, and the droplet is forced to coalesce with the aqueous stream. Typical voltages required to induce 100% coalescence range from 1.5 to 3 kV [103].

The sorting of droplets containing live cells with lactate secreting activity can be achieved on-chip by forcing confined droplets stabilized by a pH-sensitive surfactant to flow over a microfabricated trench oriented diagonally with respect to the direction of flow [99]. Droplets with low interfacial tension (low glycolytic activity) are more deformable and can exit the trench easily, while droplets with high interfacial tension (high glycolytic activity) are retained more strongly and removed at different locations.

## 4. Directed Evolution of Biomolecules in Water-in-Oil (W/O) Droplets

Compared to commercial FACS instruments that can sort only W/O/W droplets, FADS can detect both W/O or O/W droplets and double emulsions. Emulsions must be formulated to allow droplets to reliably maintain their contents during incubation and subsequent manipulation without cross contamination, coalescence, or sample loss [104]. Early research with the mineral oil- and silicon-based surfactant Abil EM 90 revealed the transfer of fluorescent markers into the continuous phase under certain conditions. The leakage of fluorophores from Abil EM 90-stabilized droplets was reduced more than 10 times by adding 5% bovine serum albumin (BSA) in the aqueous phase [105]. Fluorinated oils such as Novec HFE-7500 or FC40 in combination with fluorinated surfactants offer an alternative carrier phase to prevent crosstalk [106]. Fluorinated (or fluoro) surfactants contain a hydrophobic tail in which some carbon-hydrogen bonds are replaced with carbon-fluorine bonds [107,108]. The example of fluorosurfactants which have been successfully used for compartmentalizing both in vitro and in vivo biological systems are oligomeric perfluorinated polyethers (PFPE) coupled with polyethylene glycol (PEG) [109]. Fluorinated oils and surfactants are compatible with mammalian and bacterial cells and highly soluble to respiratory gases (oxygen and carbon dioxide). Also, they are highly compatible with PDMS and exhibit low solubility of natural organic molecules. However, crosstalk between droplets still occurs in fluorinated oils either due to the direct partitioning of biomolecules in oil or due to surfactant-mediated micellar transport [110]. 

### 4.1. Cell Compartmentalization in W/O Emulsions

**Figure 7:**
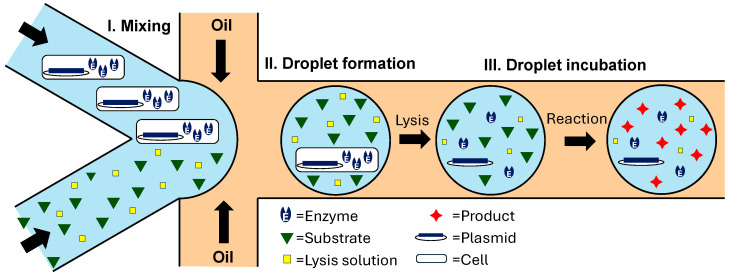
Microfluidic compartmentalization of cell lysate in W/O emulsions [111,112].

In some directed evolution experiments, in vitro transcription/translation (IVTT) solutions are used to create proteins (Figure 1d), whereas in others, cells are used as transient vehicles for gene expression (Figure 1c). In the approach shown in Figure 7, *E. coli* cells expressing the mutant enzyme library are co-compartmentalized with the cell lysis solution and fluorogenic substrate. Upon droplet formation, the cells are lysed, and the enzyme reaction takes place within the droplet, leading to the accumulation of fluorescent product. Droplets exhibiting the highest fluorescence are sorted, and plasmids are extracted and introduced into fresh *E. coli* cells for further rounds of sorting. The developed protocol allows the use of a wide variety of fluorescent substrates, regardless of their ability to penetrate the cell membrane. The procedure was used for the directed evolution of arylsulfatase from *Pseudomonas aeruginosa* (PAS) in a flow-focusing microfluidic device and allowed for a 6-fold increase in hydrolytic activity after three rounds of sorting [111,112].

**Figure 8:**
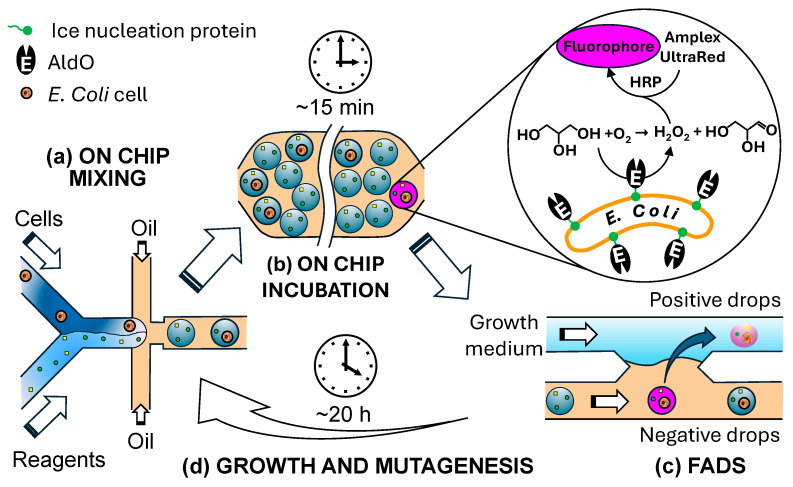
The continuous platform for the directed evolution of alditol oxidase [113].

To avoid cell lysis, the target enzyme must be either secreted or displayed on the cell surface. Rosenthal et al. [113] used an ice-nucleation protein (INP) as a surface anchor to display alditol oxidase (AldO) on the surfaces of *Escherichia coli* cells. Since the enzyme was located outside the cells, the enzymatic activity was assayed without transporting the substrate or product across the cell membrane. In this approach, targeted mutations were introduced into the gene of interest in vivo, and then a microfluidic chip was used to mix and encapsulate mutated cells with reagents, incubate the cells, and sort the best variants, as shown in Figure 8. Single cells were encapsulated in 5 pL aqueous drops containing assay reagents (Figure 8a), followed by on-chip incubation for about 15 min (Figure 8b). During that time, cells containing active AldO oxidized glycerol into glyceraldehyde and hydrogen peroxide (H_2_O_2_). The generated H_2_O_2_ was converted to a fluorescence signal by the horseradish peroxidase (HRP)-mediated conversion of Amplex™ UltraRed into a fluorescent product. Drops with a fluorescent signal above the threshold value were merged with a stream of growth medium (Figure 8c), and the recovered cells were grown for 20 h before being subjected to another round of selection (Figure 8d). Using this strategy, the catalytic activity of AldO from Streptomyces *coelicolor* was increased by >10 times with a daily throughput of >2 × 10^6^ variants, and the evolution was run continuously over several days [113].

#### Distribution of Cells in Droplets

**Figure 9:**
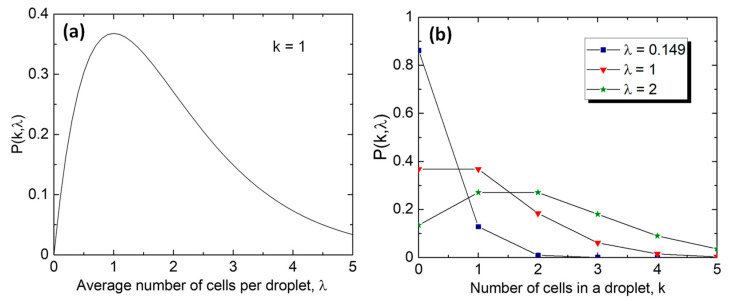
Distribution of cells in monodisperse droplets: (**a**) $P\left(\lambda ,k\right)$ vs. λ at k = 1; (**b**) $P\left(\lambda ,k\right)$ vs. k at three different λ values. Cells are randomly distributed in the feed stream.

The distribution of cells in monodisperse droplets follows the Poisson distribution [114,115]:

(4) $$P\left(\lambda ,k\right)=\frac{\lambda^{k}exp(-\lambda )}{k!}$$

where $P\left(\lambda ,k\right)$ is the proportion of droplets containing a given number of cells, k is the number of cells in a droplet, and $\lambda =CV_{drop}$ is the average number of cells per droplet, which depends on the cell concentration in the feed stream, C, and the droplet volume, $V_{drop}$. The cells are randomly distributed in the feed stream and do not approach the droplet-forming junction with a constant frequency, which is different from the droplet formation process in the dripping regime characterized by the constant droplet formation frequency. As shown in Figure 9a, the proportion of droplets loaded with a single cell is maximized when λ = 1, but then only 36.8% of all droplets will contain one cell, 36.8% of the droplets will be empty, and a further 26.4% will contain multiple cells (Figure 9b). The percentage of droplets with multiple cells can be reduced from 26.4% to 1% by decreasing λ from 1 to 0.149, but then a staggering 86.2% of all droplets will be empty and 12.8% of droplets will contain a single cell. If λ is increased from 1 to 2 by doubling the droplet volume, the proportion of empty droplets will be reduced from 36.8% to 13.5%, but 59.4% of all droplets will contain multiple cells (Figure 9b), which is even worse than at λ = 1. 

The Poisson stochastic limit of 36.8% can be overcome by the dynamic ordering of cells into a string of equally spaced cells, which can be achieved by exploiting either inertial [116,117,118,119,120] or viscoelastic [121,122,123,124] forces in the inlet microfluidic channel. In this way, the frequency of cells approaching the encapsulation area (f_cell_) can be made constant and synchronized with the frequency of droplet formation (f_drop_), i.e., f_cell_ = f_drop_, to achieve a single-cell compartmentalization.

Although the screening rates of smaller droplets are higher because they can be produced at higher frequencies in microfluidic devices, larger droplets are sometimes required to confine cells within the droplet boundaries during prolonged incubation. For example, nanolitre droplets are essential for screening the enzymatic activity of filamentous fungi because the time from which the spores of these fungi germinate to the stage where they start to display detectable enzymatic activity is ~24 h and the optimum levels of activity are reached only after several days of incubation [125]. A long incubation time, combined with the rapid growth of the fungal hyphae, makes screening filamentous fungi in picolitre droplets difficult. Indeed, hyphal tips were found to pierce the interface of 250 pl droplets in ~15 h. In contrast, if single spores were encapsulated in 18 nl droplets, the hyphal tips exited the droplets only after 32 h. Droplet puncture by hyphal tips during incubation can be suppressed by adding colloidal chitin to the dispersed phase [44].

### 4.2. In Vitro Compartmentalization (IVC) in W/O Emulsions

In vitro compartmentalization (IVC) provides the linkage of genotype to phenotype by encapsulating individual genes in W/O emulsions, enabling directed evolution of proteins and RNAs [126]. Using a completely cell-free in vitro transcription/translation (IVTT) system eliminates the need to clone DNA into cells after each round of mutagenesis, and the system is genetically simpler than cells and more prone to selection pressure [127]. Also, cell-free systems allow the expression of proteins toxic to host cells and the incorporation of non-natural amino acids and co-factors into the translated proteins. In the early studies, droplets for IVC were produced using traditional emulsification methods. However, due to the high polydispersity of droplets and variable volumes of reagents encapsulated in each droplet, the assay quality differed significantly from droplet to droplet. Using microfluidic-assisted IVC (µIVC), highly uniform droplets can be produced suitable for quantitative and comparative studies and serial operations can be performed on them. As a result, different steps of the evolution process (gene amplification, transcription/translation, and phenotypic assay) can be uncoupled, making µIVC more flexible compared to conventional IVC, where all the reagents (e.g., the PCR mixture, IVT mixture, and activity assay mixture) are present in the droplet from the beginning [51]. 

**Figure 10:**
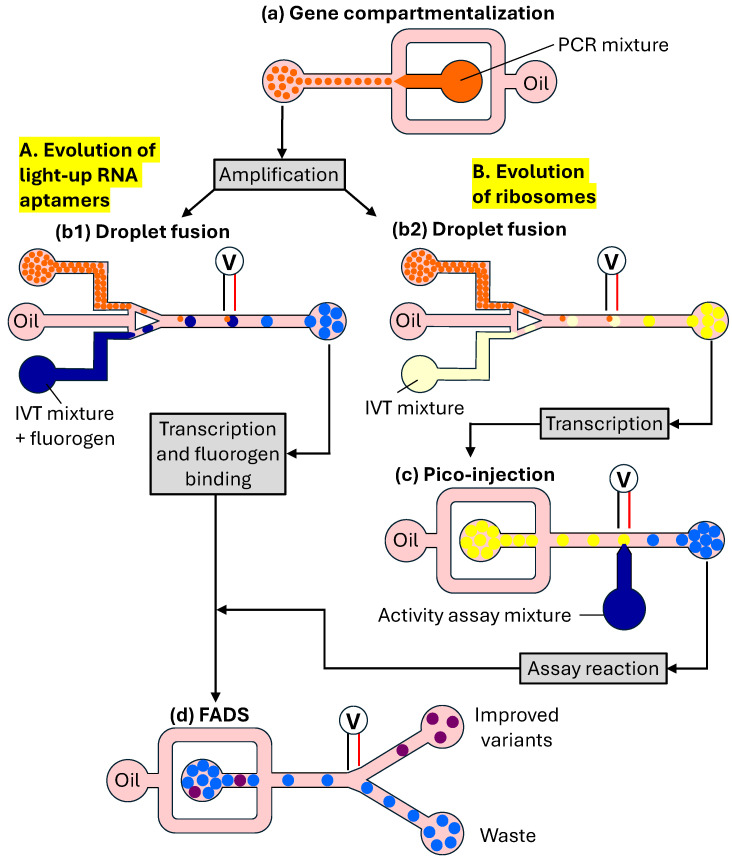
Evolution of catalytic RNAs (ribosomes) [128] and light-up RNA aptamers [129] by microfluidic-assisted in vitro compartmentalization (μIVC): (**a**) gene compartmentalization in W/O droplets; (**b1**) pairwise fusion of droplets containing amplified genes and IVT mixture/fluorogen, respectively; (**b2**) pairwise fusion of droplets containing amplified genes and IVT mixture; (**c**) picoinjection of activity assay mixture; (**d**) droplet sorting by dielectrophoretic-based FADS. Off-chip operations are shown by grey boxes.

µIVC can be used to rapidly characterize RNA molecules to validate in silico models or inform on the mutational robustness and the extent to which a sequence can be modified without altering RNA function such as catalysis or fluorogen activation. The μIVC system used to characterize the light-up RNA aptamers SRB-2 and iSpinach is shown in Figure 10A,B [128,129,130]. Light-up RNA aptamers are single-stranded RNA molecules able to specifically bind to small fluorogenic molecules and activate their fluorescence. In this workflow, DNA molecules were diluted into a PCR amplification mixture and then compartmentalized in a flow-focusing droplet microfluidic device (Figure 10a). The average number of genes per droplet, λ, was controlled by adjusting DNA dilution. Upon production, droplets were thermocycled to PCR-amplify each gene into ∼300,000 copies. Then, droplets were reinjected into a droplet fusion microfluidic device, in which they were spaced by a fluorinated oil containing 2% fluorosurfactant and synchronized one-to-one with larger droplets generated on-chip and containing an in vitro transcription (IVT) mixture and fluorogen (Figure 10b1). Pairs of droplets were fused together by an alternating current (AC) field when passing between a pair of electrodes. Upon fusion, droplets were collected and incubated to allow the genes to be transcribed and, if functional, RNA to bind the fluorogen and enable fluorescence emission. Finally, droplets were reinjected into a FADS device, in which they were spaced by a surfactant-free oil stream prior to having their fluorescence analysed (Figure 10d). Upon sorting, droplets were broken, and their DNA contents were recovered and used for the next round of screening. 

In the approach shown in Figure 10A, gene expression and the activity assay were performed simultaneously. However, uncoupling the activity assay from gene expression would make it possible to screen for activity under conditions incompatible with gene expression, as well as screen for extremely fast catalysts with reaction rates much higher than the gene expression rate [51]. The microfluidic workflow with separated gene expression and enzymatic reaction steps was used for the directed evolution of a protease to perform both steps under optimal conditions and create a viable assay [131]. Adding all reagents at once into droplets produced no measurable product, suggesting that the stepwise addition of IVTT reagents and substrate and the fine-tuning of incubation conditions in each step were indispensable.

The μIVC system for HTS of ribosomes shown in Figure 10B allows gene expression to be uncoupled from the activity assay using four microfluidic chips for the generation of DNA droplets, pairwise fusion of DNA and IVT droplets, picoinjection of activity assay reagents, and fluorescence-activated sorting of droplets [51]. First, DNA molecules were compartmentalized in droplets together with PCR reagents and a DNA intercalating dye. The droplets containing amplified DNA were distinguished from empty droplets by their fluorescence. After thermocycling, the droplets were reinjected into a second microfluidic device and paired with larger droplets containing an IVT mixture generated on-chip, and DNA/IVT pairs of droplets were coalesced using an electric field. Different concentrations of a fluorescent dye were included in the DNA and IVT droplets to control the fusion process. The emulsion was collected off-chip and incubated to allow gene transcription (DNA → RNA) to occur. The RNA-containing droplets were reinjected into the third chip to inject into each droplet an assay mixture with a high salt concentration. This allowed it to inactivate an RNA polymerase, so it did not generate high background fluorescence. After the final off-chip incubation, droplets were reinjected into a FADS chip to analyse their fluorescence. The most fluorescent droplets were broken, and the DNA was recovered, amplified by PCR, and used to start a new round of selection. Using similar microfluidic workflows, the HTS of enzymes such as protease [131], laccase [132], and β-galactosidase [133] was successfully demonstrated. 

## 5. HTS of Enzymes in Double Emulsion Droplets

W/O droplets are incompatible with downstream screening using fluorescence-activated cell sorting (FACS). This limitation of single emulsions can be resolved by performing a second compartmentalization step in which W/O droplets are emulsified in the external aqueous phase to form water-in-oil-in-water core–shell droplets that are compatible with commercial flow cytometers [134,135,136].

**Figure 11:**
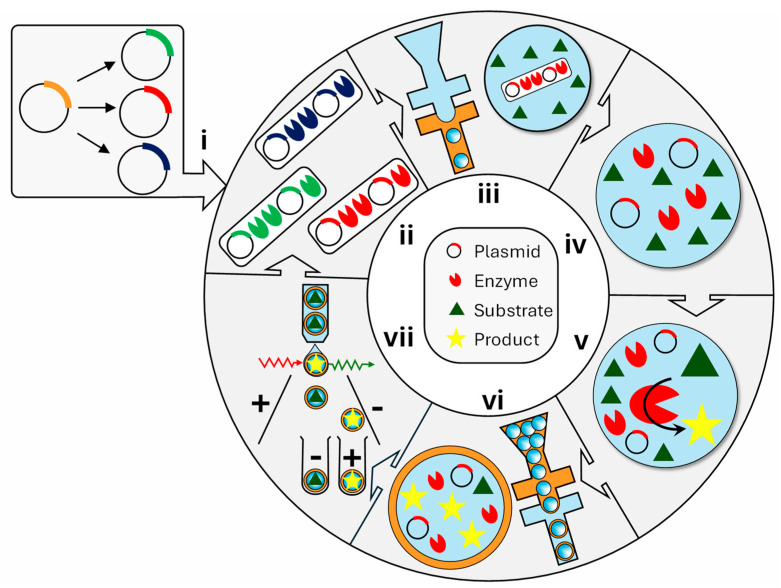
The HTS of single-cell lysate using double emulsion produced in microfluidic chips: (**i**) the generation of gene libraries from an enzyme-encoding plasmid; (**ii**) the introduction of plasmid DNA into *E. coli*; (**iii**) the compartmentalization of single cells in droplets together with substrate and lysis agents; (**iv**) cell lysis to allow enzymes to be released from the cytoplasm and react with the product; (**v**) droplet incubation for a desired period; (**vi**) the formation of double emulsions in a second microfluidic device; (**vii**) the sorting of enzyme variants in a standard flow cytometer [134].

The procedure for the HTS of single-cell lysate using double emulsion produced in two microfluidic chips is shown in Figure 11. In the first chip with hydrophobic, fluorocarbon-coated channel walls, single *E. coli* cells (ii) are compartmentalized in droplets together with substrate and lysis agents (iii). After cell lysis (iv), the substrate and expressed enzyme react to give a fluorescent product (v). The reaction is allowed to proceed for a desired period and then stopped simultaneously in all droplets by heat inactivation. After that, primary droplets are re-emulsified in a second device with the identical design (but with a hydrophilic channel coating) to form a W/O/W emulsion (vi). Variants exhibiting the highest activity are sorted in a standard flow cytometer (vii), and the recovered DNA (i) is used for further rounds of evolution [134]. Typically, a library of 10^7^ double emulsion droplets can be produced in 90 min, and their sorting takes about 15 min. The suitability of this method for directed evolution was demonstrated using arylsulfatase from *Pseudomonas aeruginosa* (PAS) as the model enzyme [134]. PAS exhibits hydrolytic activity toward the substrate fluorescein disulfate and releases fluorescein to give a fluorescent readout of reaction progress. To minimize the fraction of droplets occupied by multiple cells, the number of compartmentalized cells was 10 times smaller than the number of droplets produced, which ensured that ∼95% of occupied droplets contained a single cell. Ten minutes after compartmentalization, droplets enclosing the active PAS variant were highly fluorescent, whereas empty droplets and droplets containing a low-activity variant showed a low level of fluorescence. In the subsequent FACS sorting step, the highly fluorescent population was collected to obtain active variants. If encapsulated cells display a membrane-anchored target enzyme, cell lysis is not needed [137]. 

**Figure 12:**
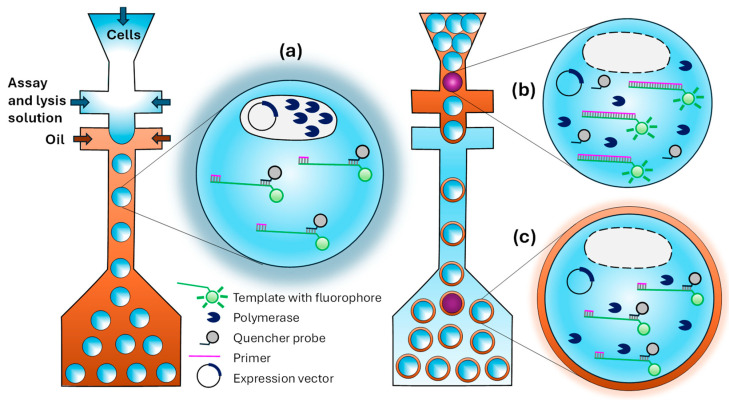
Directed evolution of polymerase using double emulsion produced in a microfluidic device: (**a**): a water-in-oil (W/O) droplet that contains a single *E. coli* cell and a fluorescence-based polymerase activity assay; (**b**) a W/O droplet containing a functional polymerase that extends a primer–template complex with RNA and releases a quencher probe; (**c**) a W/O/W droplet containing a non-functional polymerase [138].

The procedure for evolving polymerase in multiple emulsion droplets generated in microfluidic devices is shown in Figure 12. The strategy is based on using a fluorescent reporter system that produces a fluorescence signal when a primer-template complex is fully extended [138]. The reporter consists of a primer–template complex (pink and green in Figure 12) containing a downstream fluorophore that is quenched when a DNA-quencher (black) is bound to the unextended region. A library of polymerase variants is expressed in *Escherichia coli*, and single cells are encapsulated in droplets containing a fluorogenic substrate (Figure 12a). Upon lysis, the polymerase is released into the droplet and challenged to extend a primer–template complex. The DNA quencher dissociates from a primer–template complex at elevated temperatures, where thermophilic polymerases function with optimal activity and re-anneal at room temperature. Using a second flow-focusing microfluidics device, W/O droplets are emulsified into a bulk aqueous phase to generate W/O/W droplets that can be sorted using FACS. Polymerases that successfully copy a template strand into a full-length product produce a fluorescent signal by preventing a template–fluorophore pairing (Figure 12b). On the other hand, droplets remain dim in the presence of non-active polymerase when the quencher is attached to the template DNA (Figure 12c). The strategy was tested by performing a complete cycle of encapsulation, sorting, and plasmid DNA recovery when a ~1200-fold enrichment of the library in active mutants was achieved.

## 6. HTS of Biomolecules in Giant Unilamellar Vesicles

Giant unilamellar vesicles (GUVs) are spherical aqueous compartments coated by a phospholipid bilayer and dispersed in an external aqueous solution. GUVs are an excellent model system to study interactions between membrane-forming lipids and proteins. Their main advantage over liposomes is in their size, which is greater than 1 μm and comparable to the cell size, which means that GUVs can be observed directly by optical microscopy [139].

### 6.1. Microfluidic Production of Giant Unilamellar Vesicles by Phase Transfer Method

**Figure 13:**
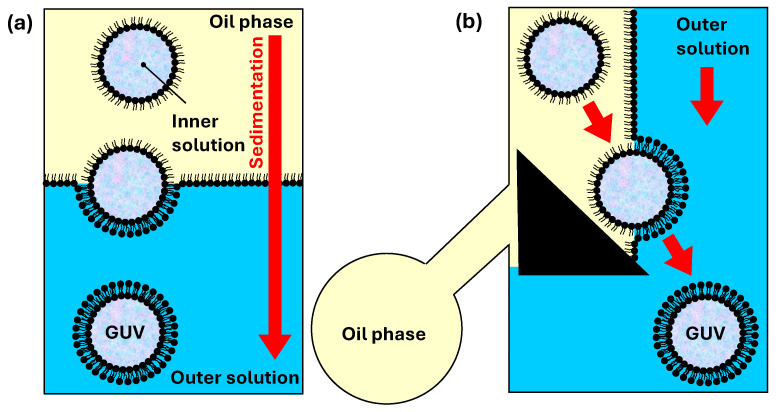
The preparation of GUVs by the phase transfer method: (**a**) The off-chip transfer of lipid monolayer-coated water-in-oil droplets from the oil phase into an outer aqueous solution by density difference. In this case, the density of the oil phase is smaller than the densities of both aqueous solutions [140]; (**b**) The on-chip transfer of droplets across the interface via a microfabricated post placed in a microchannel [144]. In both cases, lipids residing at the interface are deposited on the droplet-adsorbed lipids, forming the outer- and inner-leaflets of the membrane bilayer.

Nishimura et al. [140] performed cell-free protein synthesis inside GUVs prepared by the phase transfer method (Figure 13a). The process involves three main steps: (i) the addition of an oil–lipid mixture above the outer aqueous solution and formation of lipid monolayer at the oil/water interface; (ii) the addition of W/O emulsion with lipid-stabilized droplets to the oil phase; (iii) the transfer of lipid-monolayer-coated aqueous droplets through the interface due to density differences between the inner water phase and the oil phase and formation of GUVs in the external aqueous phase [141]. Asymmetric vesicles can be formed using different lipid compositions in the first two steps [142]. Lipid-stabilized aqueous droplets can be prepared in microfluidic devices [143], resulting in highly uniform GUVs. 

**Figure 14:**
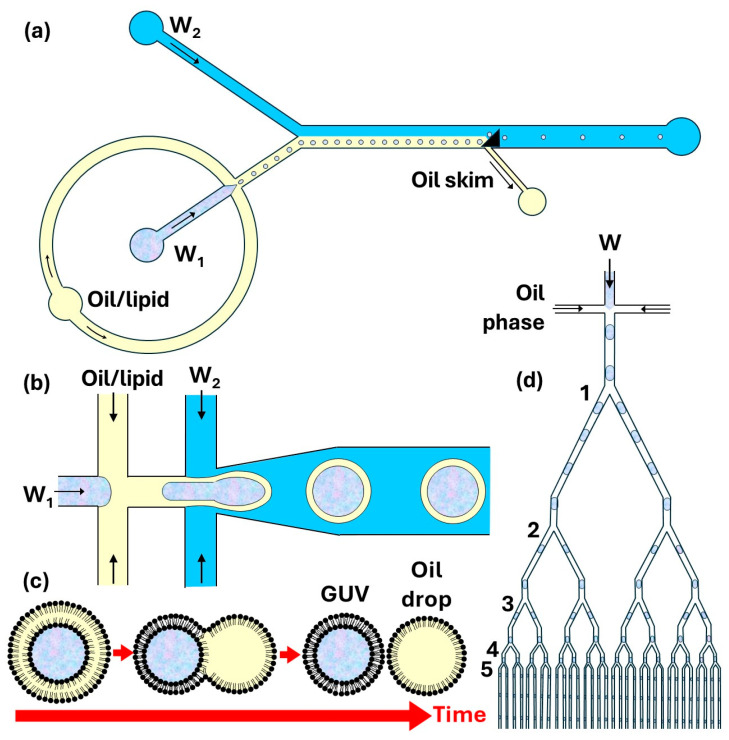
Microfluidic chips for production of GUVs: (**a**) on-chip emulsion transfer [144,145]; (**b**) double emulsion templating using two consecutive flow-focusing junctions, followed by off-chip de-wetting [147]; (**c**) de-wetting-induced formation of GUV; (**d**) flow-focusing junction, followed by a multi-V-shaped microfluidic droplet splitting channel network [148].

In the strategy shown in Figure 13b, aqueous droplets coated with a lipid monolayer are transferred across the water/oil interface by a triangular post, which leads to the deposition of a second monolayer around the droplets [144]. First, droplets that contain the inner aqueous phase (W_1_) are formed in a lipid-containing oil flow and the emulsion travels to a Y junction, where it merges with the outer aqueous stream (W_2_), as shown in Figure 14a. Due to laminar flow, a lipid-stabilized interface forms between the two co-flow streams. Finally, a triangular post mediates the phase transfer of the droplets from the oil flow into the outer aqueous flow. The excess oil/lipid solution is removed through the side channel connected to the main channel at the back of the triangular post. Asymmetric vesicles can be formed using a microfluidic device composed of a triangular post and two flow-focusing regions [145]. The on-chip formation of asymmetric GUVs includes several steps: (1) forming aqueous droplets in an oil/inner-leaflet-lipid solution; (2) replacing the inner-leaflet–lipid solution with an outer-leaflet–lipid solution; (3) forming a W/O/W double emulsion; (4) extracting excess oil/outer-leaflet–lipid solution from the double emulsion [145,146].

### 6.2. Microfluidic Production of Giant Unilamellar Vesicles by Double Emulsion Templating

The double emulsion templating method involves the formation of a lipid-stabilized water-in-oil-in-water (W/O/W) double emulsion using a PDMS device (Figure 14b) [147] or glass capillary device [149], followed by oil removal from the shell by extraction into the outer aqueous phase and subsequent evaporation [149] or by de-wetting, as shown in Figure 14c [150]. During oil extraction, the oil shell becomes progressively thinner causing the lipid monolayers on inner and outer interface to fuse together and form a lipid bilayer membrane. If the middle phase contains an oil which is poorly soluble in water, such as oleic acid, ethanol can be added to the outer aqueous phase to increase oil solubility [151]. To minimize the amount of oil that needs to be removed from the middle phase, W/O/W emulsion droplets with ultrathin (submicron) shells can be formed [152,153].

The oil phase can be separated from the inner phase by de-wetting, which is a much faster process than solvent extraction and typically takes less than 10 min [150]. The de-wetting process can occur on-chip [150] or off-chip [154]. The oil phase spontaneously de-wets to produce separate oil droplets and GUVs if the interfacial tensions satisfy the following inequalities [154]:

(5) $$\gamma_{1}-\left(\gamma_{2}+\gamma_{3}\right)<0$$

(6) $$\gamma_{2}-\left(\gamma_{3}+\gamma_{1}\right)<0$$

(7) $$\gamma_{3}-\left(\gamma_{1}+\gamma_{2}\right)>0$$

where $\gamma_{1}$ is the interfacial tension between the internal and external aqueous phase (W_1_ and W_2_), $\gamma_{2}$ is the interfacial tension between the oil–lipid phase and external aqueous phase (O and W_2_), and $\gamma_{3}$ is the interfacial tension between the oil–lipid phase and internal aqueous phase (O and W_1_). GUVs with multiple compartments are prepared using template multiple emulsion droplets consisting of several inner droplets [155,156]. 

One of the most effective oils used in the middle phase is 1-octanol because the presence of short 1-octanol molecules (8 carbon atoms) among much longer lipid molecules (~18 carbon atoms) is energetically unfavourable as it forms defects in the ordered structures of lipids [150]. As a result, the lipid-bilayer tension increases considerably in the presence of 1-octanol, pushing 1-octanol molecules and excess lipid molecules out of the shell and into the growing oil drop, Figure 14c. Ultimately, the bilayer zips along the entire interface, forming a GUV and pinching off an octanol droplet. The resultant mixture of octanol droplets and GUVs can be separated based on density difference between water and octanol. Usually, a poloxamer 188 (Pluronic F-68) surfactant was added to both aqueous phases to fine-tune the interfacial tensions and the de-wetting process [150]. A high lipid concentration in octanol facilitates spontaneous de-wetting and allows the production of GUVs without the need for any surfactant [154]. The surfactant-free production of GUVs is promoted by implementing a serpentine channel downstream of the droplet-forming junctions, which imposes additional shear force on the double emulsion droplets and assists in fast advective removal of oil [147]. 

### 6.3. Microfluidic Production of GUVs by Fusion of Internally Trapped SUVs

**Figure 15:**
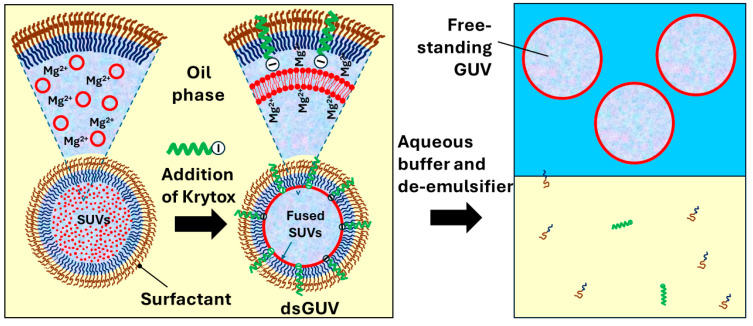
The formation of GUVs by the charge-induced fusion of internally entrapped small unilamellar vesicles (SUVs) at the surfactant-loaded droplet interface [157].

Another microfluidic method for the formation of GUVs is based on charge-induced fusion of small unilamellar vesicles (SUVs) entrapped within water-in-oil (W/O) droplets stabilized by a commercial fluorosurfactant [157]. First, a flow-focusing PDMS device is used to encapsulate SUVs in W/O droplets. After that, the charge-driven adhesion of positively charged SUVs to the surfactant-loaded droplet interface is triggered by the incorporation of anionic fluorosurfactant Krytox to the surfactant layer, as shown in Figure 15. A positive charge of SUVs is imparted by the presence of Mg^2+^ ions in the buffer solution within the droplets. As a result of SUV fusion at the droplet periphery, droplet-stabilized GUVs (dsGUVs) are formed, consisting of a spherical lipid bilayer that is encapsulated within a surfactant-stabilized W/O droplet. The sequential loading of various sub-cellular functional units into the dsGUVs is achieved by microfluidic picoinjection [152]. Free-standing GUVs are released from the dsGUVs by pipetting an iso-osmotic buffer solution on top of the emulsion and adding the de-emulsifier perfluoro-octanol to displace the surfactant from the interface. Using this technique, GUVs with positively or negatively charged lipid membranes can be prepared to investigate GUV–cell interactions [148]. 

Standard flow-focusing junctions have been used to prepare dsGUVs with diameters of 20–50 µm [157,158]. dsGUVs with diameters of ~3 μm were produced in a Y-shaped bifurcating downstream channel by splitting each parent W/O droplet loaded with SUVs through five consecutive division steps (1 → 2 → 4 → 8 → 16 → 32) into 32 equally sized daughter droplets [148], as shown in Figure 14d. A similar droplet splitting strategy was used to produce >10^6^ monodisperse droplets in several minutes for digital PCR by splitting ~13 nL parent droplets 8 times into 256 equally sized 50-picolitre daughter droplets [159]. A symmetric division of liposomes in a microfluidic channel was accomplished by flowing a suspension of liposomes against the sharp edge of a wedge-shaped splitter [160].

## 7. Directed Evolution in Microgels

**Figure 16:**
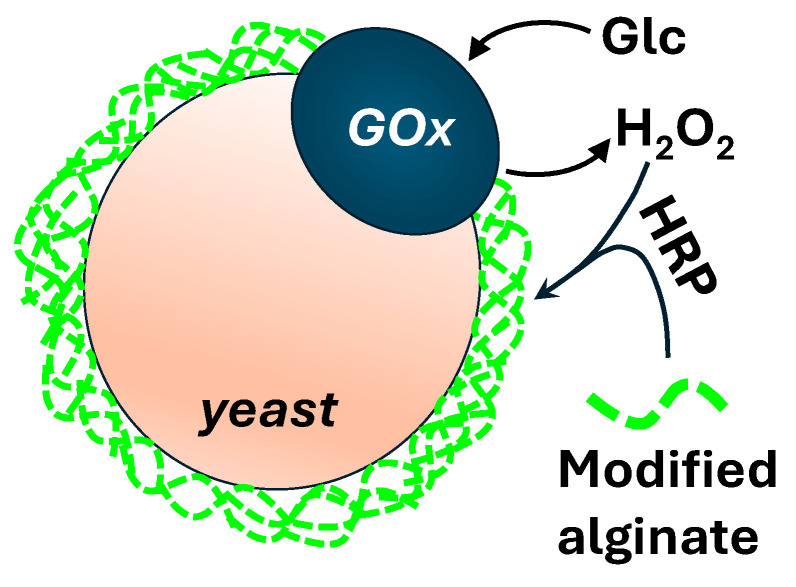
A directed evolution strategy for screening glucose oxidase (GOx) enzyme variants in a homogeneous solution. GOx expressed on the yeast surface triggers the crosslinking of alginate grafted with phenol moieties and aminofluorescein using hydrogen peroxide in the presence of horse radish peroxidase (HRP) and glucose (Glc) [163].

Microgel-based screening strategies enabling the use of FACS can be based on: (a) localized enzyme-mediated crosslinking of fluorescent polymer at the cell surface without droplet compartmentalization [161]; (b) trapping an enzyme, its encoding DNA, and the fluorescent product in microgels formed within emulsion droplets [162]. In the first approach, glucose oxidase (GOx) displayed on the outer cell walls of yeasts is used to initiate a cascade reaction with horseradish peroxidase (HRP), resulting in the peroxidase-mediated crosslinking of fluorescent alginate carrying phenol moieties, as shown in Figure 16. 

The second approach consists of generating aqueous droplets in which single cells are encapsulated together with a dissolved gellable polymer, fluorogenic substrate, and lysis agent. Following cell lysis and droplet incubation for enzymatic catalysis, the gellable polymer is crosslinked to transform droplets into gel microbeads. Depending on the polymer’s nature, the gel network can be formed upon lowering the temperature, with the addition of an oppositely charged crosslinker, or due to covalent bonding [164]. A typical gel-forming polymer used in this technique is agarose, which undergoes sol–gel phase transition from random coils to double helices upon lowering the temperature. The helices further aggregate to form interconnected bundles held together by hydrogen bonds. Standard agarose has a gelling temperature of 34–38 °C. To prevent gelling during droplet generation and incubation, agarose with an ultra-low gelling temperature (8–17 °C) was used [165,166,167]. Once solidified, the beads formed from this type of agarose remain solid all the way up to 56 °C. Agarose droplets can be solidified off-chip in an ice bath [168] or by trapping droplets of agarose solution on-chip and reducing the chip temperature below the gelling temperature [169]. Monodisperse agarose beads produced by droplet microfluidics have been used for detecting cytokine secretion, phenotypical maturation, the proliferation of single, activated Jurkat T cells [166], and screening lipid production of *Lipomyces starkey* yeast cells [170]. 

**Figure 17:**
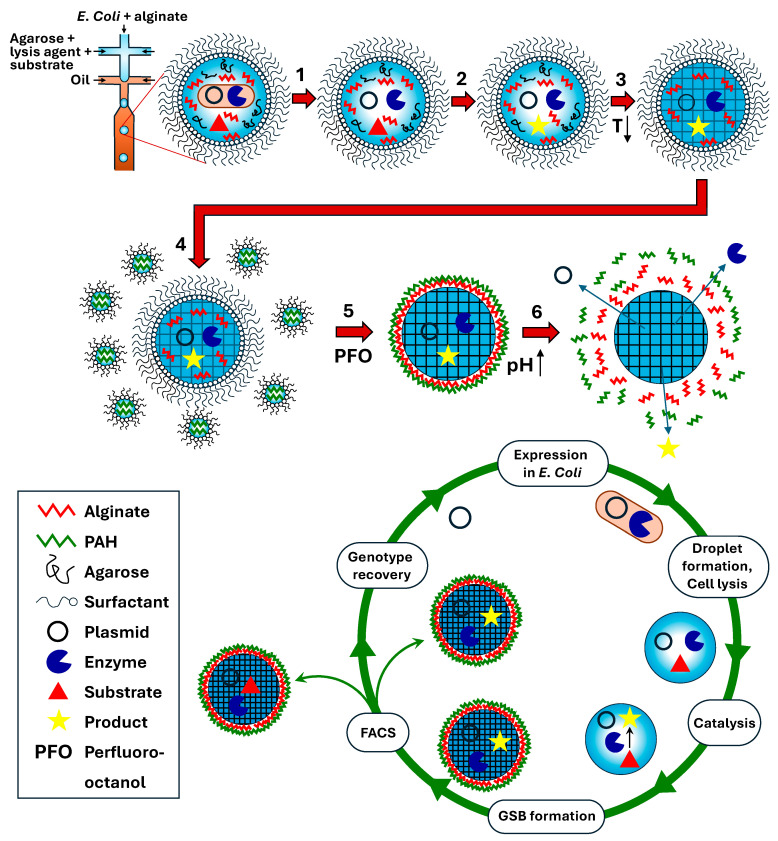
Directed evolution in (gel core)-(polyelectrolyte shell) (GSB) beads: (1) Cel lysis; (2) Enzymatic reaction (3) Droplet gelation upon cooling; (4) Mixing gel beads with PAH-containing W/O emulsion; (5) Surfactant displacement and polyelectrolyte film formation; (6) Shell decomposition at high pH and the release of microgel content [162].

A thin polyelectrolyte shell can be built around agarose beads by layer-by-layer (LbL) self-assembly to produce gel shell beads (GSBs) with stimuli-responsive release properties, as shown in Figure 17 [162]. Using this approach, polydisperse W/O emulsion droplets that contain the polycation PAH (poly(allylamine hydrochloride)) are mixed with alginate-entrapping agarose beads. Then, perfluoro-octanol (PFO) is added to displace the surfactant layer from the microgel surface and expose negatively charged carboxylate groups of alginate to positively charged NH_3_^+^ groups of PAH. To decrease the molecular weight cut-off of the formed polyelectrolyte membrane, the gel particles can be additionally coated with the polyanion PSS (poly(sodium styrene sulfonate) but must have diameters of up to ~50 μm to be sorted by FACS [164]. The shell can be dissolved using buffer solutions with a high ionic strength or with a pH > pKa of PAH to recover the coding plasmid. The system can be used to perform the directed evolution of a phosphotriesterase and isolate a 20-fold faster mutant in less than one hour [162]. 

## 8. HTS in Core–Shell Gel Microcapsules

In this approach, single cells are encapsulated within core–shell droplets, followed by shell gelation and capsule incubation and screening. The gel network is usually formed by the photopolymerization of UV-curable monomers, such as N-isopropylacrylamide and acrylamide, or photocrosslinkable polymers, such as gelatine methacryloyl (GelMA) and poly(ethylene glycol) diacrylate (PEGDA). In addition to photocrosslinkable monomer or polymer, the shell material must contain a photoinitiator, which decomposes when exposed to UV or visible light to initiate free radical polymerization. Visible light photoinitiation is advantageous due to damaging effects of UV radiation on DNA. Typical photoinitiators that can be activated by visible (blue) light are camphorquinone (CQ), eosin Y, riboflavin, and lithium phenyl (2,4,6-trimethylbenzoyl) phosphinate (LAP) [171].

**Figure 18:**
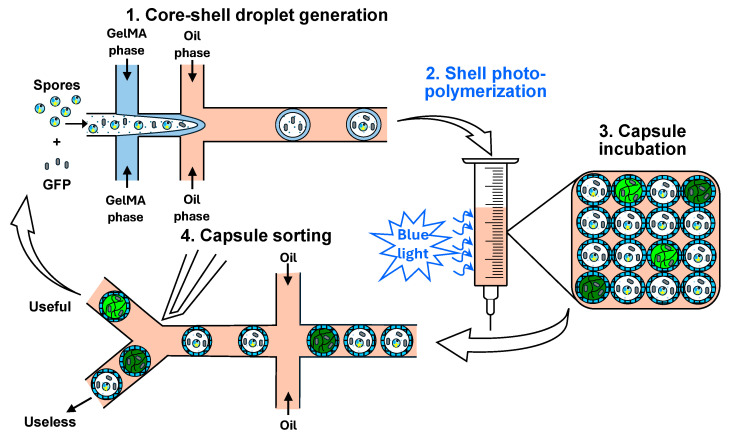
Microfluidic HTS of filamentous fungi within core–shell microcapsules consisting of aqueous cores and hydrogel shells [172].

Recently, core–shell GelMA microcapsules fabricated by droplet microfluidics were used for the encapsulation, culture, and HTS of filamentous fungi for the purpose of optimizing their protein production [172]. The crosslinked GelMA shell acted as a shield to prevent the hyphae from puncturing the droplets during growth and sustain prolonged culture. Double emulsion droplets were generated in a flow-focusing microfluidic device composed of two consecutive cross junctions, as shown in Figure 18. The inner aqueous phase was composed of a spore suspension, the GelMA phase was a buffer solution containing 8% GelMA and 0.5% LAP photoinitiator, and the oil phase was a fluorocarbon oil containing 2% surfactant. The generated droplets were exposed to blue light to crosslink GelMA precursor. The incubated droplets were injected into the sorting chip and sorted by FADS at a frequency of 6–10 Hz. Target droplets with high fluorescences were electrically deflected into the collecting channel, while droplets with fluorescences below the screening threshold were discarded into the waste channel. The high biocompatibility of the capsules was verified by encapsulating *A. niger* spores, which maintained protein synthesis and secretion for a long time. 

## 9. Conclusions

The compartmentalization of cells and genes in emulsion droplets is a powerful approach to creating millions of independent microreactors for the screening of biomolecules. Droplet microfluidic chips allow the production of monodisperse droplets at ultrahigh throughput, as well as the dynamic ordering of cells into equally spaced strings, thus enabling the rapid creation of assays for the single-cell screening of biological diversity. Furthermore, a precise sequential addition of multiple reagents into pre-formed droplets can be achieved using microfluidic picoinjectors at kilohertz rates. Controlled reagent volumes can also be introduced by pairwise droplet fusion in microfluidic channels passively or actively using local electric field. In addition, droplets can be incubated on-chip for a controlled time in delay lines, incubation chambers, and microwells and can be reinjected after incubation to be sorted by dielectrophoresis or electrocoalescence. To achieve better resemblance of droplet compartments with cellular structures, droplets can be converted into giant lipid vesicles by replacing conventional surfactants from the interface by a lipid bilayer. Also, the stability and structural integrity of droplets during prolonged incubation can be preserved by encapsulating droplets within a thin biocompatible gel layer to form core–shell capsules with aqueous core and biomimetic shell. 

Using microfluidics for in vitro compartmentalization (IVC), millions of genes in a library can be individually compartmentalized in monodisperse aqueous droplets, and serial operations can be performed on them. This allows the different steps of the evolution process (gene amplification, transcription, and phenotypic assay) to be uncoupled, making the method highly flexible. The process was successfully tested for the selection and evolution of a variety of catalytic and fluorogenic RNAs, DNAs, and proteins. 

The conversion of water-in-oil (W/O) emulsion droplets into water-in-oil-in-water (W/O/W) emulsion droplets enables droplet sorting by FACS. The screening procedure based on W/O/W emulsions consists of the encapsulation of single cells and substrates in W/O droplets, cell lysis (unless the encapsulated cells display a membrane-anchored target enzyme), an assay reaction, a second emulsification step in a separate microfluidic chip, and the sorting of double emulsions using a standard cytometric sorter. Alternatively, FACS sorting can be achieved by solidifying W/O emulsion droplets into gel microbeads, which can be coated with a polyelectrolyte multilayer. 

Traditional microfluidics is based on using monolithic chips that can perform multiple operations. However, monolithic chips lack operational and structural flexibility and are difficult to operate and reconfigure. In the future, the concept of modular microfluidics may be used more widely to increase the adoption of microfluidics in mainstream biomedical research [173]. Modular microfluidics is based on using standardized chips for individual fluidic operations that can be designed, fabricated, and tested separately and easily assembled into a customized, re-configured microfluidic platform [173,174,175].
